# Integrating a Physical Therapy Program into Usual Care for Hospital Inpatients with Major Depressive Disorder: Findings from a Case Series

**DOI:** 10.3390/healthcare14131848

**Published:** 2026-06-25

**Authors:** José Lesmes Poveda-López, Juan Francisco Roy, Bárbara Marco-Gómez, Ana Villagrasa-Cantín, Sara Pérez-Mansilla, Raquel Lafuente-Ureta, Carolina Jiménez-Sánchez

**Affiliations:** 1 Department of Physical Therapy, Faculty of Health Sciences, Universidad San Jorge, 50830 Villanueva de Gállego, Zaragoza, Spain; jlpoveda@usj.es (J.L.P.-L.); cjimenez@usj.es (C.J.-S.); 2 Department of Psychology, Faculty of Health Sciences, Universidad San Jorge, 50830 Villanueva de Gállego, Zaragoza, Spain; jfroy@usj.es; 3 Faculty of Health Sciences, Universidad Internacional de La Rioja, 26006 Logroño, La Rioja, Spain; 4 Department of Psychiatry, Royo Villanova Hospital, Health Service of Aragón, Universidad de Zaragoza, 50009 Zaragoza, Zaragoza, Spain; barbaramarcogomez@gmail.com (B.M.-G.); avillagrasac@salud.aragon.es (A.V.-C.); sara_1697@hotmail.es (S.P.-M.)

**Keywords:** Major Depressive Disorder, physical therapy modalities, quality of life, mental health services

## Abstract

**Background/Objectives:** Major Depressive Disorder (MDD) is a leading cause of disability, yet physical therapy (PT) is underrepresented in hospital-based psychiatric care. While exercise is a known adjunctive treatment, specific evidence on functional, task-oriented interventions in acute settings remains scarce. This study explored changes in quality of life, depressive symptoms, pain, and self-efficacy in patients with MDD following a specialized hospital-based PT program focused on functional movement and autonomy. **Methods:** We conducted a prospective pre–post case series in the Short-Stay Psychiatric Unit of the Royo Villanova University Hospital (Zaragoza, Spain). We recruited seven adult patients with MDD via convenience sampling. The intervention consisted of a group-based PT program (two 45 min sessions/week during the hospital stay) utilizing task-oriented functional exercises targeting progressive strength, balance, and motor control designed to enhance self-efficacy through activities of daily living (ADLs), combined with health education. Outcomes included the EQ-5D-3L (quality of life), MADRS (depression), NRS (pain), GSE (self-efficacy), and GCPC-UN-ESU (satisfaction). **Results:** All seven participants (100%) exhibited a positive upward trend in self-perceived health status via the EQ-VAS (mean increase of 35 points). Six cases (85.7%) showed preliminary positive trends in the anxiety/depression dimension of the EQ-5D-3L, with the mean Single Index Value increasing from 0.310 to 0.683. Reductions in depressive symptom severity were observed in six participants, with several transitioning toward moderate or mild levels. Additionally, four patients reported descriptive reductions in pain intensity and showed favorable shifts in self-efficacy scores. Six participants expressed high satisfaction with the intervention. **Conclusions:** Integrating a hospital-based functional PT program with standard care may offer preliminary benefits for quality of life and reduce depressive symptoms in MDD patients. These findings suggest that task-oriented PT presents a feasible complementary approach for acute psychiatric admissions, although larger controlled trials are needed to confirm these exploratory results.

## 1. Introduction

Mental health is a fundamental human right and a cornerstone of community development, enabling individuals to navigate life stressors through personal resilience and functional abilities [1]. According to the World Health Organization, the erosion of mental well-being leads to severe disorders characterized by profound distress, functional impairment, and an escalating burden on healthcare services [2]. Among these conditions, Major Depressive Disorder (MDD) stands out as a prevalent mood disorder defined by profound sadness and a sustained loss of interest lasting at least two weeks [3,4]. MDD is the most common cause of disability worldwide, affecting women and individuals with lower economic income more severely [5,6].

Globally, over 300 million individuals live with a depressive disorder, representing approximately 4.4% of the world’s population [7]. In Europe, this pathology imposes a massive economic burden, estimated at €118 billion annually [8]. This public health challenge is highly pronounced in Spain, where depressive disorders represent a leading cause of psychiatric morbidity, with an estimated prevalence of 4% to 8% across the general adult population [9]. Reflecting the substantial burden of this condition, national epidemiological data indicate that prevalence rates can reach up to 30% to 50% in specific vulnerable cohorts, positioning depression as a primary focus within institutional mental health care [10,11,12]. This demographic is particularly vulnerable as there is a reciprocal clinical correlation between depressive disorders and somatic pain. Chronic health issues often serve as potent precursors to depressive episodes; conversely, psychological distress frequently somatizes, increasing pain sensitivity and worsening clinical outcomes for physical pathologies [13]. This bidirectional relationship reinforces the need for multidisciplinary approaches in treating MDD, especially in aging populations where comorbidity is prevalent.

MDD is a complex, multifactorial disease influenced by both genetic and environmental factors [14,15]. From a physiological perspective, it is characterized by a decrease in key neurotransmitters—serotonin, norepinephrine, and dopamine [16,17]—often mediated by the hyperactivity of the hypothalamic–pituitary–adrenal axis. This dysfunction triggers a cascade of effects, including the reduction in hippocampal volume [18] and a systemic inflammatory state evidenced by increased pro-inflammatory cytokines in the blood. This aligns with the inflammatory hypothesis of depression, which posits that chronic low-grade inflammation disrupts the blood–brain barrier and impairs synaptic plasticity [19]. Consequently, patients often experience not only psychological symptoms like anhedonia and anergia but also a significant physical phenotype of depression, including chronic pain, muscle atrophy, and reduced functional capacity [16,19].

Standard treatments for MDD typically combine pharmacological and psychological interventions [20]. While effective, these therapies can be compromised by a lack of patient adherence or adverse side effects [21]. Physical therapy (PT) offers a promising yet underutilized complementary cornerstone in psychiatric care [22]. While general physical activity encompasses any bodily movement that increases energy expenditure, and exercise therapy represents structured, planned activities designed to improve fitness, physical therapy distinctly integrates these elements within a clinical framework [23]. In this professional context, physical therapists are experts in addressing movement-related problems and can enhance the “body–mind” connection through tailored, prescriptive therapeutic exercise and health education [24]. By increasing patients’ awareness of their physical capabilities, PT can reduce pain, strengthen the body, and improve mood through the release of endorphins, the stimulation of brain-derived neurotrophic factor and the promotion of neuroplasticity [14]. In this regard, recent high-level evidence supports the effectiveness of structured exercise as a key adjunctive treatment, highlighting the specific capacity of specialized physical therapy to precision-target both the biological and psychosocial dimensions of mental disorders [25].

While there is currently substantial interest within mental health physical therapy regarding exercise for treating depression [26,27] and broad epidemiologic evidence supports the efficacy of standard exercise doses in reducing MDD symptoms [28], the majority of research to date has focused on aerobic or standardized resistance protocols within outpatient settings [29]. Crucially, traditional exercise paradigms often fail when translated into acute psychiatric wards. Previous inpatient studies frequently report low adherence and high drop-out rates [30], as standard repetitive training demands a level of intrinsic motivation and physical energy that acutely hospitalized patients [31], severely affected by anhedonia and psychomotor retardation, simply cannot muster [20]. Furthermore, conventional fitness equipment is often unfeasible or restricted within locked psychiatric units due to safety protocols and spatial limitations [32,33].

This case series distinguishes itself by focusing on the clinical application of functional exercise within an inpatient setting, specifically designed to enhance participants’ self-efficacy [34]. Unlike traditional protocols, the interventions described herein utilize task-oriented movements that mimic daily activities. By shifting the therapeutic target from physical fitness parameters toward the immediate reclamation of basic functional autonomy, this approach lowers the psychological barrier to entry for patients in crisis [31,34]. In the wider context of mental health physical therapy, specialized approaches such as Basic Body Awareness Therapy (BBAT) have demonstrated clinical value as adjunctive treatments for major depression [35]. However, implementing BBAT in acute hospital settings remains challenging, particularly within the constraints of short-stay inpatient units. This underscores the need to explore more accessible, task-oriented physical therapy approaches that can be readily implemented in standard inpatient care environments. By targeting the intersection of physical competence and psychological empowerment, this study provides a unique perspective on how functional movement serves as a bridge to restoring autonomy in acutely depressed patients, a granular aspect often overlooked in large-scale clinical trials.

Despite its potential, structured PT is rarely integrated into acute, short-stay psychiatric units, where the sedentary nature of hospitalization may actually worsen physical and affective symptoms. To address this gap in the literature, the primary objective of this case series was to explore the potential impact of a novel hospital-based functional physical therapy program—combining task-oriented therapeutic exercise and health education—on health-related quality of life in acutely hospitalized patients with Major Depressive Disorder. Additionally, this study aimed to observe secondary clinical trends in depressive symptoms, pain intensity, and self-efficacy, while also assessing participant satisfaction with the proposed intervention. Herein, we present the preliminary and exploratory findings from the initial cohort of seven participants integrated into this acute inpatient care pathway.

## 2. Materials and Methods

### 2.1. Study Design and Ethical Considerations

We conducted a case series (7) to evaluate the preliminary effects of a combined PT intervention program based on therapeutic exercise and health education. The study design, detailed procedures, and eligibility criteria were previously published in a formal peer-reviewed protocol [36]. The trial was prospectively registered at ClinicalTrials.gov (NCT06983405). Although this study is reported as a case series, it is part of a larger registered study, and the present manuscript provides a descriptive analysis of the initial cases included prior to completion of the full study. The Ethics Committee of Universidad San Jorge (CETICA No. 38/3/24-25) and the Research Ethics Committee of Aragón (CEICA C.I. PI25/233) approved the study. All participants provided written informed consent prior to inclusion. This case series adhered to the CARE 2013 guidelines [37] for clinical case reporting.

### 2.2. Participants and Recruitment

Patients diagnosed with MDD were recruited using consecutive sampling. Participants were selected from those admitted to the acute inpatient psychiatry unit at Royo Villanova Hospital (Zaragoza, Spain) between May 2025 and February 2026. During the study period, 17 potential participants underwent eligibility screening. Eight patients declined participation. Given the acute distress and vulnerability of patients in an acute psychiatric ward, the clinical team prioritized the therapeutic alliance and ethical care over rigorous questioning regarding study refusal. Direct inquiries were deliberately avoided to prevent patients from feeling pressured or fearing that declining would disappoint their clinicians. Therefore, reasons for non-participation were clinically inferred by the psychiatric team based on patients’ spontaneous feedback and clinical presentations.

**Figure 1:**
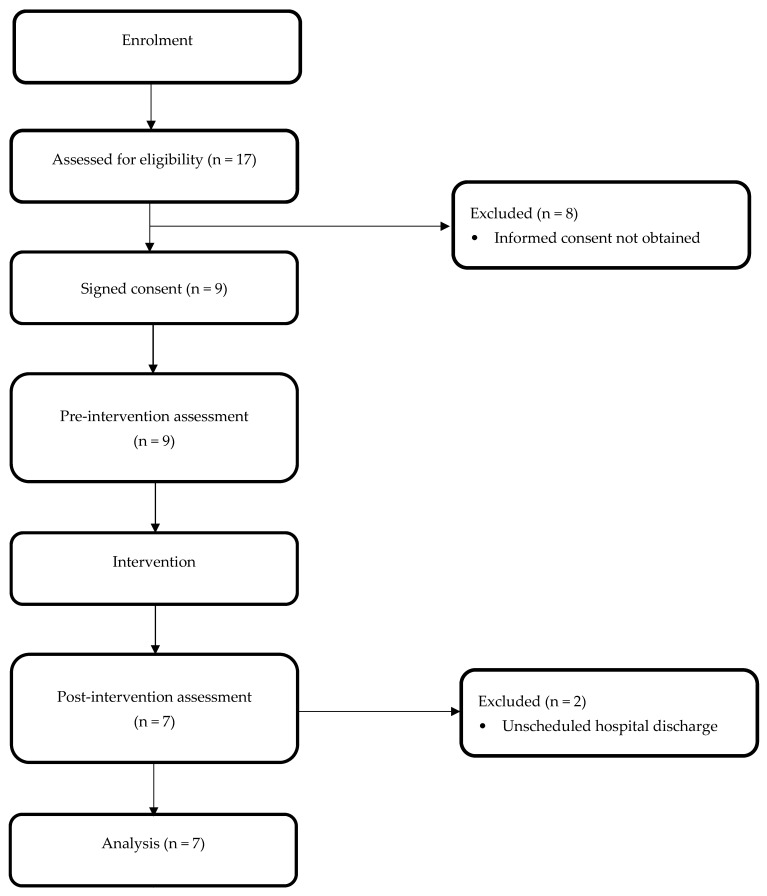
Flow chart.

The primary barriers identified were core symptoms of severe MDD, such as profound apathy and clinophilia, which severely limited patients’ motivation to engage. Additionally, a subgroup of patients exhibited marked suspicion and paranoia—often associated with severe depression with psychotic features—or a generalized opposition to the hospitalization itself. Furthermore, several patients expressed a cognitive distortion or belief that participating in the research might artificially prolong their hospital stay for administrative or investigative reasons. The unfounded nature of this concern is evidenced by the fact that two enrolled participants were later discharged as soon as they were clinically ready, prioritizing their return home over the completion of the post-intervention research assessments. Recruitment was facilitated by the treating psychiatric team. Prior to enrollment, candidates were informed that the intervention—a physical therapy program comprising exercise and education sessions—served as a clinical adjunct to improve physical health and quality of life. Participants were briefed on the program duration and assured of the strict confidentiality of all collected data. To ensure ethical integrity, it was explicitly communicated to all potential participants that their decision to join or decline the study was strictly voluntary and would have no impact on their medical treatment, hospital stay, or discharge planning. Inclusion and exclusion criteria remained strictly as defined in the original protocol. Initially, nine participants were enrolled; however, two were lost to follow-up due to unscheduled hospital discharges that precluded post-intervention assessments. Consequently, seven participants were included in the final analysis. The detailed selection and recruitment process, including the reasons for exclusion, is illustrated in the flow chart presented in Figure 1.

### 2.3. Intervention

Participants received a multi-component intervention consisting of functional therapeutic exercise and health education. The physical therapy intervention was delivered by a licensed physiotherapist with 24 years of clinical experience. The therapist possessed specific professional training in task-oriented functional exercise and therapeutic movement management, without holding formal postgraduate international certifications in mental health-specific physical therapy frameworks. The PT intervention was integrated into the standard care pathway of the short-stay psychiatric unit. Inpatients were referred by the medical team as part of routine clinical practice, and the physical therapist delivered the intervention as an integrated component of the unit’s overall treatment program. However, research data were only collected from participants who met the eligibility criteria and provided written informed consent. The sessions were group-based and included other inpatients with diverse psychiatric conditions. The program was designed to consist of two 45 min sessions per week over a maximum hospitalization period of three weeks (up to 6 sessions). Each 45 min session followed a highly structured, three-phase therapeutic sequence: a warm-up phase, a core conditioning phase, and a cool-down phase.

The session commenced with a 5-to-10 min warm-up focusing on active general joint mobility. This was followed by the core conditioning phase (30 min), which was evenly divided into resistance and balance components. First, 15 min were dedicated to resistance training utilizing body weight or elastic bands to replicate functional activities of daily living; this included upper-limb pushing and pulling movements, lower-limb squats and deadlifts, and abdominal strengthening exercises, all strictly tailored to each patient’s individual physical capacities. Second, 15 min were allocated to static and dynamic balance training in a standing position, which involved reducing the base of support and performing tandem or semi-tandem gait displacements to enhance stability and prevent falls.

For both the resistance and balance components, training volume was standardized at 2 to 3 sets of 8 to 12 repetitions per exercise [38]. Exercise intensity was closely monitored and tailored using the Borg Rating of Perceived Exertion (RPE) scale [39], targeting a light-to-moderate perceived effort (Borg RPE scores between 2 and 4) [40]. Progression was individualized and criteria-based; the physical therapist progressed the exercise difficulty (e.g., increasing elastic band resistance, increasing repetitions, or transitioning from stable to unstable balance surfaces) only when a participant could comfortably complete the current sets and repetitions with proper technique and without experiencing excessive fatigue or exacerbation of physical/psychiatric symptoms.

Finally, the session concluded with a 5-to-10 min cool-down phase dedicated to progressive muscle relaxation techniques paired with deep, mindful diaphragmatic breathing to reduce physical tension. The health education component promoted self-management and treatment adherence through discussions on the biological and psychological links between physical activity and mental health, training in mood self-monitoring techniques, and information regarding community resources to maintain an active lifestyle following hospital discharge. The physical therapist individualized the progression of the exercises according to each participant’s perceived exertion and fatigue levels.

### 2.4. Variables and Outcome Measures

Study outcomes were assessed at pre-intervention (upon hospital admission) and at post-intervention (at hospital discharge). To minimize detection bias, clinical measurements were performed by a blinded team—comprising a psychiatrist and two mental health nurse specialists—who were not involved in the intervention delivery or the daily clinical care of the participants. All data were pseudonymized to ensure strict participants’ confidentiality and compliance with ethical standards, maintaining the integrity of the blinded assessment process throughout the study duration.

In alignment with the pilot nature of this study, the primary outcome was health-related quality of life, evaluated through the EuroQol-5D-3L (EQ-5D-3L) [41]. The prioritization of a generic health-related quality of life measure over depression-specific scales is justified by the multidimensional impact of Major Depressive Disorder (MDD). MDD entails profound cognitive and functional impairments that severely disrupt an individual’s capacity to manage daily life [33,42]. Modern mental health research emphasizes that tracking global health perception and patient-centered outcomes represents a major methodological strength, capturing vital clinical benefits and functional recoveries that traditional symptom-severity scales often overlook [30]. Furthermore, given that physical activity concurrently modulates both mental and physical health domains—such as mobility or pain—a generic instrument like the EQ-5D-3L is uniquely suited to monitor these interconnected components in depressed patients undergoing supervised exercise [8,43], aligning with established clinical evaluation standards in both primary and specialized care [10]. This instrument includes a five-dimensional descriptive system—covering mobility, self-care, usual activities, pain/discomfort, and anxiety/depression—where each dimension is rated on three levels of severity: (1) no problems, (2) some problems, and (3) extreme problems. The resulting health state is interpreted as a five-digit code, which can be converted into a single index value, where higher scores represent better health states. To interpret clinical significance, a change of 0.03–0.05 in the index value is considered the Minimal Clinically Important Difference (MCID) [44]. Furthermore, changes exceeding 0.10, 0.20, and 0.30 are classified as moderate, large, and very large improvements, respectively. Additionally, the tool incorporates the EuroQol Visual Analogue Scale (EQ-VAS), a 0–100 scale specifically designed to capture the participant’s own perception of their overall health status, where 0 represents the worst and 100 the best imaginable health.

Secondary outcomes addressed the program’s preliminary clinical effects through a battery of validated instruments. Depressive symptomatology was measured using the Montgomery–Åsberg Depression Rating Scale (MADRS) [45], with total scores ranging from 0 to 60; higher scores indicate greater severity, where standard cut-offs define 0–6 as normal/recovered and scores above 34 as severe depression. To interpret the clinical relevance of symptom reduction, the MCID was benchmarked against current methodological consensus, which establishes an MCID range of 3 to 9 points for moderate-to-severe depression, with a reduction of ≥6 points serving as a robust threshold for definitive clinical improvement [46].

Pain intensity was assessed via the Numerical Rating Scale (NRS) [47] on a 0–10 point scale, where 0 represents ‘no pain’ and 10 ‘the worst imaginable pain’. To establish the clinical relevance of changes in pain perception, a reduction of 1.0 to 1.5 points was utilized as the benchmark for the MCID, based on recent evidence in chronic pain populations [48].

Perceived self-efficacy was evaluated through the General Self-Efficacy Scale (GSE) [49], where higher cumulative scores reflect a stronger belief in one’s ability to respond to novel or difficult situations. Due to the current lack of a validated, anchor-based MCID for this scale in psychiatric populations, the clinical relevance of self-efficacy improvements was interpreted using the Smallest Detectable Change (SDC). Following established psychometric benchmarks, a threshold of >4.33 points was utilized to ensure that individual score changes reflected true clinical improvement beyond potential measurement error [50].

Furthermore, intervention acceptability was recorded post-intervention using the GCPC-UN-ESU survey [51], a 19-item tool measuring treatment satisfaction across four dimensions on a 5-point Likert scale; in this instrument, higher values indicate superior levels of patient satisfaction and treatment acceptability.

### 2.5. Data Analysis

Data analysis was primarily descriptive, given the exploratory nature of this case series and the small sample size (n = 7). Individual changes from baseline to post-intervention were calculated and reported for each case, allowing the visualization of individual variability and clinical trends. When appropriate, pre–post differences were expressed as absolute and percentage changes in outcome measures. Percentage change was calculated as ((post-intervention value − baseline value)/baseline value) × 100. No inferential statistical tests were performed, as the study was not designed to assess effectiveness or establish causal relationships.

All analyses were conducted using standard statistical software (IBM SPSS Statistics (version 30; IBM Corp., Armonk, NY, USA), and results are presented at both individual and aggregate levels to facilitate clinical interpretation.

## 3. Results

### 3.1. Sociodemographic and Clinical Characteristics

The study sample comprised seven participants, predominantly female (85.7%), with a mean age of 51.6 ± 18.1 years (range: 25–75). Anthropometric data (weight and BMI) were obtained for six participants, as these metrics could not be recorded for Case 7 due to patient refusal related to a comorbid eating disorder. For the recorded cases, the mean weight was 78.6 ± 19.2 kg and the mean Body Mass Index (BMI) was 29.9 ± 6.3 kg/m^2^, indicating a trend toward obesity within the group. All participants were receiving pharmacological treatment at the time of the intervention. The demographic and clinical characteristics of the participants are detailed in Table 1, while their specific pharmacological treatments at study entry and during hospitalization are described in Table 2.

### 3.2. Case Presentations

Case 1

A 47-year-old female completed the six physical therapy sessions. Post-intervention, her EQ-5D-3L dimensions showed that mobility remained at level 1, self-care changed from level 2 to 1, and usual activities, pain/discomfort, and anxiety/depression shifted from level 3 to 2. This corresponded to an index value change from −0.049 to 0.754 (+0.803) and an EQ-VAS score increase from 0 to 55 points. Regarding clinical scales, the MADRS score decreased from 46 to 12 points, the pain NRS dropped from 8 to 2 points, and the GSE score changed from 10 to 14 points. Finally, the patient assigned a score of 5 across all satisfaction survey dimensions.

Case 2

A 75-year-old female with a medical history of essential hypertension, dyslipidemia, mild segmental ischemic colitis, gout, and major depression completed the six physical therapy sessions. Post-intervention, her EQ-5D-3L mobility, self-care, and usual activities remained at level 1, while pain/discomfort changed from level 2 to 1, and anxiety/depression shifted from level 3 to 1, resulting in an index value increase from 0.452 to 1.000 (+0.548) and an EQ-VAS increase from 40 to 70 points. The MADRS total score decreased from 37 to 28 points, with the reported sadness subscore changing from 5 to 0. The pain NRS decreased from 5 to 3.5, and the GSE score moved from 18 to 22 points. The patient recorded a score of 5 across all satisfaction survey dimensions.

Case 3

A 65-year-old female with a medical history of essential hypertension, hyperferritinemia, and hepatic steatosis completed the six physical therapy sessions. Post-intervention, her EQ-5D-3L scores for mobility, self-care, and pain/discomfort remained at level 1, usual activities remained at level 2, and anxiety/depression changed from level 3 to 2. Her index value changed from 0.470 to 0.843 (+0.373) and the EQ-VAS score increased from 20 to 60 points. The MADRS total score decreased from 40 to 14 points, with the lassitude and suicidal thoughts subscores reaching 0 points. No changes were recorded on the pain NRS, which remained at 0 points. The baseline GSE score was 20 points, and the post-intervention assessment was not completed. She recorded a score of 5 across all satisfaction survey dimensions.

Case 4

A 37-year-old male with a history of substance use disorder (cocaine) completed two physical therapy sessions. Post-intervention, all EQ-5D-3L dimensions (mobility, self-care, usual activities, pain/discomfort, and anxiety/depression) remained at level 1, leaving the index value stable at 1.0. The EQ-VAS score increased from 85 to 95 points. The MADRS total score changed from 4 to 0 points, and the pain NRS remained at 0 points. The GSE score increased from 19 to 35 points. Finally, the patient recorded a score of 5 across all satisfaction survey dimensions.

Case 5

A 44-year-old female with no significant comorbidities completed two physical therapy sessions. Post-intervention, her EQ-5D-3L dimensions showed that mobility changed from level 2 to 1, self-care remained at level 1, and usual activities shifted from level 3 to 1, while both pain/discomfort and anxiety/depression decreased from level 3 to 2. This corresponded to an index value increase from −0.021 to 0.825 (+0.846) and an EQ-VAS score increase from 20 to 65 points. Regarding clinical scales, the MADRS score decreased from 41 to 26 points, the pain NRS dropped from 7 to 0 points, and the GSE score increased from 10 to 25 points. Finally, her post-intervention evaluation reflected a mean satisfaction score of 3.89.

Case 6

A 68-year-old female with a medical history of essential hypertension completed two physical therapy sessions. Post-intervention, her EQ-5D-3L descriptive system showed that mobility changed from level 2 to 1, while pain/discomfort and anxiety/depression both decreased from level 3 to 2; conversely, self-care changed from level 1 to 2, and usual activities moved from level 1 to 3. This corresponded to an index value change from 0.174 to 0.205 (+0.031) and an EQ-VAS score increase from 0 to 35 points. Regarding clinical scales, the MADRS total score moved from 30 to 31 points, though the suicidal thoughts subscore decreased from 2 to 0 points. The pain NRS decreased from 7 to 5 points, and the GSE score increased from 12 to 19 points. Finally, her post-intervention evaluation reflected a mean satisfaction score of 3.42.

Case 7

A 25-year-old female with a medical history of eating disorders, obesity, and autoimmune primary hypothyroidism completed two physical therapy sessions. Post-intervention, her EQ-5D-3L descriptive system showed that mobility changed from level 2 to 1 and anxiety/depression shifted from level 3 to 2, while both self-care and usual activities remained at level 2. This corresponded to an index value change from 0.141 to 0.157 (+0.016) and an EQ-VAS score increase from 0 to 30 points. Regarding clinical scales, the MADRS total score moved from 36 to 34 points, the pain NRS changed from 5 to 6 points, and the GSE score moved from 16 to 17 points. Finally, her post-intervention evaluation reflected a mean satisfaction score of 4.26.

### 3.3. Clinical Outcomes and Preliminary Effects

All participants (100%) reported an increase in EQ-VAS scores, with a mean increase of 35 points (from a baseline mean of 23.6 to 58.6 post-intervention). Regarding the EQ-5D-3L descriptive dimensions, mobility improved in three participants (42.9%), and the anxiety/depression dimension decreased in severity for six participants (85.7%). The pain/discomfort dimension improved in four cases (57.1%), while the usual activities domain showed improvement in two participants (28.6%). Conversely, Case 6 reported an increase in severity levels for both self-care and usual activities. Individual case results, including pre- and post-intervention scores, differences, and the EQ-5D-3L Single Index Values, are summarized in Table 3.

In terms of depressive symptomatology, six out of seven participants (71.4%) recorded a reduction in total MADRS scores. Pain intensity, measured via the NRS, decreased in four out of the five participants (80%) who reported pain at baseline. Regarding self-efficacy (GSE), scores increased in several instances, such as Case 4 (from 19 to 35) and Case 5 (from 10 to 25); however, Case 3 did not complete the post-intervention assessment for this scale. Participant satisfaction, measured by the GCPC-UN-ESU survey, reached a mean score of 4.65 out of 5 across the sample. Individual scores for depression, pain intensity, and self-efficacy, including pre- and post-intervention values and their respective differences, are presented in Table 4.

### 3.4. Satisfaction and Perception

Participant satisfaction, measured via the GCPC-UN-ESU survey, was remarkably high. Four participants (Cases 1 through 4) provided maximum scores (5/5) across all evaluation items. The remaining participants reported mean satisfaction scores of 3.89 (Case 5), 3.42 (Case 6), and 4.26 (Case 7). These results reflect a generally very positive perception of the PT program, highlighting its perceived benefit in the management of their condition.

## 4. Discussion

This case series provides preliminary exploratory evidence on the potential clinical value of integrating a structured PT program into the usual care of inpatients with MDD. Improvements were observed in health-related quality of life, depressive symptoms, pain intensity, and self-efficacy in several participants, suggesting that PT may serve as a meaningful complementary component within acute psychiatric settings.

A key finding of this study is the overall improvement in perceived health status, with all participants showing higher EQ-VAS scores after the intervention. Beyond the subjective perception captured by the EQ-VAS, the EQ-5D-3L index value also reflected positive trends. Most participants (Cases 1, 2, 3, 5, and 6) had values in the range of approximately 0.03–0.05, which are consistent with the magnitude of minimally important differences reported in the literature for the EQ-5D [44]. Notably, several cases showed ‘large’ or ‘very large’ improvements (increases >0.20 or 0.30), suggesting that the structured PT program may be associated with meaningful improvements in health-related quality of life, even in short-term acute psychiatric stays. These findings should be interpreted with caution, as the absence of a control group precludes causal attribution and the observed improvements may also reflect pharmacological treatment, hospitalization, spontaneous recovery, or regression to the mean.

However, these results align with previous research demonstrating that exercise-based interventions can enhance subjective well-being and overall quality of life in individuals with depression. Meta-analyses have shown that physical activity produces moderate improvements in quality of life across mental health populations, likely mediated by increased physical functioning, social engagement, and neurobiological adaptations [52,53]. In addition, previous research also indicates that educational interventions targeting health and lifestyle in individuals with mental health conditions can contribute to improvements in perceived health status, particularly through enhanced health literacy and behavioral change [54].

Reductions in depressive symptoms, as measured by the MADRS, were observed in five of the seven participants, with several cases demonstrating changes that can be considered clinically meaningful. These results are in line with previous evidence supporting exercise as an effective adjunctive treatment for depression. Exercise has been shown to modulate the hypothalamic–pituitary–adrenal axis, increase brain-derived neurotrophic factor, and promote neuroplasticity—mechanisms that may contribute to mood improvement [25,52,55].

A fundamental in the PT program is resistance exercise, which not only improves functional capacity but may also influence biological processes described in previous research. Evidence suggests that muscle contraction during strength training may stimulate the release of myokines such as irisin, which has been proposed to cross the blood–brain barrier and be associated with neurobiological effects. These pathways have been linked to mechanisms such as the modulation of BDNF expression and potential neuroprotective effects [42,56]; however, these variables were not assessed in the present study, and their role in the observed improvements remains hypothetical for this specific sample. Although causality cannot be inferred due to the study design, the magnitude of improvement observed in several cases may suggest a potential contribution of PT to symptom reduction.

Pain, a frequently overlooked but highly prevalent symptom in MDD, also improved in most participants who reported baseline pain. This is clinically relevant, as chronic pain and depression share bidirectional pathways involving inflammation, central sensitization, and reduced physical activity [57]. Similar patterns have been described in people with severe mental illness, where musculoskeletal pain and somatic complaints are highly prevalent and often under-treated, contributing to poorer functioning and quality of life [58]. In this context, exercise and physiotherapy-based interventions have shown promising effects in reducing pain and improving physical functioning among individuals with schizophrenia and other SMI diagnoses, suggesting that structured movement-based programs can address both physical and psychological symptom burden [16,59]. PT interventions, integrating targeted movement, strengthening, and body awareness strategies such as BBAT, may therefore help disrupt this cycle by improving mobility, reducing somatic tension, and potentially alleviating pain-related disability in patients with MDD and other severe mental disorders [60].

Self-efficacy outcomes showed greater heterogeneity, yet notable improvements were observed in some cases. Self-efficacy is a recognized mediator of the mental health benefits of exercise, influencing motivation, perceived control, and resilience [61]. Recent evidence highlights that enhancing self-efficacy is a key mechanism through which physical activity improves depressive symptoms, functional capacity, and overall well-being in individuals with mood disorders [52,59]. In populations with MMD, exercise-based interventions have similarly been shown to increase self-efficacy, which in turn predicts greater adherence, improved physical functioning, and reductions in psychiatric symptom severity [62]. Similarly, the observed increase in self-efficacy aligns with prior evidence suggesting that health education and self-management approaches improve individuals’ confidence in managing their condition, especially in populations with chronic conditions [63]. Enhancing self-efficacy during hospitalization may therefore be particularly valuable, as it can support engagement in recovery-oriented behaviors after discharge and facilitate long-term maintenance of physical activity, which is often challenging for individuals with MDD due to low motivation, fatigue, and cognitive condition. These findings suggest that PT programs delivered in acute psychiatric settings may be relevant for exploring potential effects on self-efficacy, although their role in sustained improvements beyond the inpatient stay remains to be determined. In this context, functional outcomes also showed variability. Case 6, in particular, presented a non-linear trajectory, with a decline in the self-care domain after the intervention. This finding highlights the clinical heterogeneity of acute psychiatric inpatients, in whom symptomatic improvement and functional recovery do not necessarily follow parallel pathways. Considering the participant’s complex pharmacological regimen and comorbid chronic pain, these factors may have contributed to transient difficulties in self-care; however, this interpretation remains speculative and is not directly supported by the available data. PT can prompt patients to recognize their actual physical limitations more realistically, which should be interpreted not as a therapeutic failure, but as a clinical phase of recovery and re-adjustment.

Participant satisfaction was high across the sample, with multiple individuals rating the intervention at the maximum score. This reinforces the acceptability and perceived usefulness of integrating PT into psychiatric inpatient care. The group-based format of the sessions, which were open to all inpatients within the unit, may have also contributed to these positive perceptions through social interaction and shared engagement. Therefore, the observed benefits may reflect not only the effects of therapeutic exercise but also the supportive social context of the program, which has been recognized as an important component of exercise-based interventions in mental health [40]. Although our sample size is small, the absence of adverse events underscores the safety of implementing such programs even during the acute phases of psychiatric hospitalization. This is consistent with previous studies reporting low risk associated with supervised exercise in psychiatric populations [64].

Despite these promising findings, several limitations must be acknowledged. The small sample size and lack of a control group limit the generalizability of the results and preclude causal inference. Variability in intervention dosage—four participants completed only two sessions due to early, clinically driven hospital discharge—may have influenced the magnitude and interpretation of the observed changes. Given the exploratory nature of this preliminary case series, the loss of two participants is acknowledged as a limitation, as it may have influenced the observed trends. Outcomes were assessed only at discharge, limiting conclusions about whether improvements were maintained over time, which is particularly relevant in MDD. Given the descriptive nature of this case series, the improvements observed should be interpreted with caution, as they may also be explained by concurrent standard psychiatric care, pharmacological treatment, spontaneous recovery, or hospitalization effects. Potential changes in medication during admission were not systematically monitored, and the timing of baseline assessment in relation to hospital admission and early substance abstinence may also have influenced the observed outcomes, collectively acting as confounding factors. Future studies should explore whether early responses are associated with long-term adherence following hospital discharge. Moreover, randomized controlled designs with larger samples and more standardized intervention protocols are needed to better evaluate the role of PT in acute psychiatric settings and explore continuity of care after discharge. Additionally, future studies should incorporate post-discharge follow-up strategies to assess long-term adherence and maintenance of physical activity in the community. The inclusion of physical functioning outcome measures in future studies may also help to better capture the broader impact of exercise interventions beyond psychological outcomes. Furthermore, incorporating patient perspectives through qualitative approaches in future studies would provide a more comprehensive understanding of the intervention’s impact.

Nevertheless, this study contributes novel preliminary evidence to a field where PT remains underrepresented. The positive trends observed suggest that integrating PT into short-stay psychiatric units appears feasible, safe, and potentially beneficial within this small sample. Further investigation is warranted to determine optimal intervention components, dosing, and long-term outcomes in controlled studies.

## 5. Conclusions

This case series suggests that integrating a structured PT program based on therapeutic exercise and health education into acute psychiatric inpatient units is both feasible and safe for individuals with MDD. Preliminary findings suggest an improvement in self-perceived overall health status, alongside reductions in depressive symptomatology and pain intensity in the majority of participants. Furthermore, the observed increases in self-efficacy levels and the high satisfaction rates reported may indicate the potential value of physical therapy as a complementary intervention within the multidisciplinary care of acute MDD.

Despite the limitations inherent to the small sample size and study design, these findings should be interpreted as exploratory and hypothesis-generating. They highlight the importance of addressing the physical phenotype of depression during hospitalization. Future research is warranted to confirm these clinical benefits, determine the optimal intervention dosage, and evaluate the long-term sustainability of these outcomes following hospital discharge.

## Figures and Tables

**Table 1:** Demographic and clinical participant characteristics.

Case	Sex	Age (Years)	Weight (kg)	BMI (kg/m^2^)	Clinical History/Comorbidities
1	Female	47	78.5	31.1	No significant comorbidities reported
2	Female	75	69.0	28.7	HT, Dyslipidemia, Ischemic Colitis, Gout
3	Female	65	61.7	24.7	HT, Hyperferritinemia, Hepatic Steatosis
4	Male	37	91.5	27.9	Substance use disorder (cocaine)
5	Female	44	60.5	25.2	No significant comorbidities reported
6	Female	68	110.2	42	Essential hypertension
7	Female	25	-	-	ED, obesity, Hypothyroidism
Mean (SD)	-	51.6 (18.1)	78.6 (19.2)	29.9 (6.3)	-

Note: BMI: Body Mass Index; ED: eating disorders; HT: hypertension; kg: kilograms; m^2^: square meters; SD: Standard Deviation. Participant data were collected upon admission to the acute inpatient psychiatry unit.

**Table 2:** Concomitant medication profiles of the included cases.

Case	Medication and Dosage at Admission	Administration/ Frequency at Admission	Medication at Discharge	Dosage Changes
1	Diazepam 2.5–2.5–5 mg	+5 mg repeatable if needed	Same baseline regimen preserved	Stable
	Lormetazepam 2 mg	0–0–1 (at bedtime)		
	Fluoxetine 20 mg	1–0–0 (Breakfast)		
	Sertraline 25 mg	1–1–0 (Breakfast–Lunch)		
	Trazodone 100 mg	0–0–1 (At bedtime)		
	Gabapentin 100 mg	1–1–1 (Breakfast–Lunch–Dinner)		
	Paracetamol 500 mg	As needed		
2	Lisinopril 20 mg	1 tablet/24 h	Venlafaxine 225 mg, Lamotrigine 100 mg, Quetiapine 300 mg, Clonazepam 1 mg.	Optimization: Significant reduction in antidepressant load (Venlafaxine reduced from 375 mg to 225 mg). Quetiapine titrated upwards for stabilization.
	Simvastatin 20 mg	1 tablet/24 h		
	Rivotril (Clonazepam) 1 mg	0–0–1 (Dinner)		
	Lamotrigine 100 mg	0–0–1 (At bedtime)		
	Quetiapine 300 mg	0–0–1 (Dinner)		
	Venlafaxina 375 mg	1–0–0 (Breakfast)		
	Hidroferol (Calcifediol)	1 tablet monthly		
3	Desvenlafaxine 50 mg	1–0–0 (Breakfast)	Regimen adjusted during stay.	Clonazepam dosage was dynamically optimized based on acute anxiety management.
	Rivotril (Clonazepam)	0.5–0.5–0.5–1 mg		
	Aripiprazole 30 mg	0–1–0 (Lunch)		
	Risperidone 6 mg	0–0–1 (At bedtime)		
	Lamotrigine	50–0–100 mg		
	Lisinopril/Hydrochlorothiazide 20/12.5 mg	1 tablet/24 h		
4	Desvenlafaxine 100 mg	1–0–0 (Breakfast)	Regimen optimized (Desvenlafaxine adjusted).	Maintained on stable antidepressant baseline with dynamic clinical optimization.
	Lurasidone 37 mg	0–1–0 (Lunch)		
	Trazodone 100 mg	0–0–1 (At bedtime)		
	Clonazepam 0.5 mg	As needed		
5	Desvenlafaxine 100 mg	1–0–0 (Breakfast)	Regimen optimized (Desvenlafaxine adjusted).	Monitored and cross-tapered safely to target clinical stabilization.
	Sertraline 50 mg	0–1–0 (Lunch)		
	Trazodone 200 mg	0–0–1 (At bedtime)		
	Lormetazepam 2 mg	0–0–1 (At bedtime)		
	Quetiapine 100 mg	0–0–1 (At bedtime)		
	Clonazepam 2 mg	1/2–1/2–1/2–1/2 tablet		
	Dexketoprofen 25 mg	As needed for pain		
	Vitamin B12	A week		
6	Duloxetine 30 mg	1–1–0 (Breakfast–Lunch)	Regimen optimized (Quetiapine adjusted to 100 mg).	Optimization: Reduction in Quetiapine dosage achieved prior to discharge.
	Fluoxetine 20 mg	1–1–0 (Breakfast–Lunch)		
	Clonazepam 0.5 mg	1–1–1 (Breakfast–Lunch–Dinner)		
	Quetiapine 150 mg	2 tablets at bedtime		
	Tramadol/Paracetamol 37.5/325 mg	Every 8 h as needed for pain		
	Ixia (Olmesartan) 40 mg	1–0–0 (Breakfast)		
	Omeprazole 20 mg	1–0–0 (Breakfast)		
7	Folic acid 5 mg	1–0–0 (Breakfast)	Regimen optimized (Lormetazepam adjusted).	Sleep and anxiety medication dynamically tailored to improve tolerance.
	Zonisamide 150 mg	1–0–0 (Breakfast)		
	Fluoxetine 60 mg	1–0–0 (Breakfast)		
	Diazepam 2.5 mg	1–0–0 (Breakfast)		
	Lormetazepam 2 mg	0–0–1 (At bedtime)		
	Trazodone 50 mg	0–0–1 (At bedtime)		

Note: h: hours; mg: milligrams; tablet/24 h: one tablet every 24 h. Participant data reflect the stable concomitant pharmacological treatment regimens at the time of study enrollment.

**Table 3:** Comparison of EQ-5D-3L dimensions and EQ-VAS pre- and post-Intervention.

Case	Mobility	Self-Care	Usual Activities	Pain/ Discomfort	Anxiety/ Depression	Single Index Value	EQ-VAS (0–100)
	Pre/Post	Pre/Post	Pre/Post	Pre/Post	Pre/Post	Pre/Post (Diff.)	Pre/Post (Diff.)
1	1/1	2/1	3/2	3/2	3/2	−0.049/0.754 (+0.803)	0/55 (+55)
2	1/1	1/1	1/1	2/1	3/1	0.452/1 (+0.548)	40/70 (+30)
3	1/1	1/1	2/2	1/1	3/2	0.470/0.843 (+0.373)	20/60 (+40)
4	1/1	1/1	1/1	1/1	1/1	1/1 (0)	85/95 (+10)
5	2/1	1/1	3/1	3/2	3/2	−0.021/0.825 (+0.846)	20/65 (+45)
6	2/1	1/2	1/3	3/2	3/2	0.174/0.205 (+0.031)	0/35 (+35)
7	2/1	2/2	2/2	2/3	3/2	0.141/0.157 (+0.016)	0/30 (+30)
Mean	1.4/1.0	1.3/1.3	1.9/1.7	2.1/1.7	2.7/1.7	0.310/0.683 (+0.373)	23.6/58.6 (+35)

Note: EQ-5D-3L: EuroQol 5-Dimensions 3-Levels; EQ-VAS: EuroQol Visual Analogue Scale; Diff.: Difference. For the five dimensions, scores range from 1 (no problems) to 3 (extreme problems). The Single Index Value was calculated using the Spanish value set, where 1 represents perfect health and values below 0 represent states worse than death. Data are presented as pre-intervention/post-intervention (Difference).

**Table 4:** Clinical outcomes, self-efficacy and patient satisfaction.

Case	MADRS (0–60)	Δ MADRS	NRS (0–10)	Δ NRS	GSE (10–40)	Δ GSE	Satisfaction
	Pre/Post	(%)	Pre/Post	(%)	Pre/Post	(%)	(0–5)
1	46/12	−73.9%	8.0/2.0	−75.0%	10/14	+40.0%	5.00
2	37/28	−24.3%	5.0/3.5	−30.0%	18/22	+22.2%	5.00
3	40/14	−65.0%	0.0/0.0	0.0%	20/N/A	—	5.00
4	4/0	−100.0%	0.0/0.0	0.0%	19/35	+84.2%	5.00
5	41/26	−36.6%	7.0/0.0	−100.0%	10/25	+150.0%	3.89
6	30/31	+3.3%	7.0/5.0	−29.0%	12/19	+58.3%	3.42
7	36/34	−5.6%	5.0/6.0	+20.0%	16/17	+6.2%	4.26
Mean	33.4/20.7	−38%	4.6/2.6	−43.5%	15/22	+60.2%	4.51

Note: MADRS: Montgomery–Åsberg Depression Rating Scale (range 0–60); NRS: Numeric Rating Scale for pain (range 0–10); GSE: General Self-Efficacy Scale (range 10–40); Diff.: Difference; N/A: Not Available. For MADRS and NRS, a reduction in scores indicates clinical improvement; for GSE, an increase indicates improvement. Data are presented as pre-intervention/post-intervention (Difference).

## Data Availability

The data presented in this study are available on request from the corresponding author. The data are not publicly available due to ethical and privacy restrictions, as they contain sensitive clinical information from a small cohort of psychiatric inpatients.

## Abbreviations

The following abbreviations are used in this manuscript:

PT	Physical Therapy
MDD	Major Depressive Disorder
EQ-5D-3L	EuroQol 5-Dimensions 3-Levels
EQ-VAS	EuroQol Visual Analogue Scale
MADRS	Montgomery–Åsberg Depression Rating Scale
NRS	Numerical Rating Scale
GSE	General Self-Efficacy Scale
GCPC-UN-ESU	Global Care Patient Satisfaction with Nursing Students’ Care
CETICA	Comité de Ética en Investigación de la Universidad San Jorge
CEICA	Comité de Ética de la Investigación de la Comunidad de Aragón
CARE	CAse REport Guidelines
ED	Eating Disorders
MCID	Minimal Clinically Important Difference
SD	Standard Deviation
BMI	Body Mass Index
